# Fusion to chicken C3d enhances the immunogenicity of the M2 protein of avian influenza virus

**DOI:** 10.1186/1743-422X-7-89

**Published:** 2010-05-09

**Authors:** Zhenhua Zhang, Yongqing Li, Shufang Xu, Fuyong Chen, Li Zhang, Beiyu Jiang, Xiaoling Chen

**Affiliations:** 1 College of Animal Medicine, China Agricultural University, Beijing 100094, China; 2 Institute of Animal Husbandry and Veterinary Medicine, Beijing Academy of Agricultural and Forestry Sciences, Beijing 100097, China

## Abstract

**Background:**

Current vaccines to avian influenzae virus (AIV), a highly contagious disease of birds, need to be constantly updated due to the high level of variation in the target antigens. Therefore, a vaccine that could induce broad cross protection against AIV is required. The M2 membrane protein is structurally conserved amongst AIV subtypes but tends in induce a poor immune response, whereas C3d has been shown in many species to enhance immunogenicity. In this study, we investigated the potential of M2-avian C3d fusion proteins to provide effective immunity.

**Results:**

We fused chicken complement C3d to sM2 (M2 protein with the transmembrane region deleted) of AIV and expressed four fusion proteins, GST (Glutathione S-transferase tagged proteins in pGEX expression vector) -C3d-sM2, GST-C3d-L2-sM2, GST-C3d-L1-C3d-sM2 and GST-C3d-L1-C3d-L2-sM2 were used to immunize mice. In addition, Specific pathogen free (SPF) chickens were inoculated with the plasmids pcDNA-sM2, pcDNA-C3d-L1-C3d-L2-sM2, GST-sM2 and GST-C3d-L1-C3d-L2-sM2. The immune response was monitored by an enzyme-linked immunosorbent assay (ELISA) for sM2 antibody, and all the test animals were challenged with A/chicken/Bei Jing/WD9/98 (H9N2) virus. Results revealed that the anti-sM2 antibody in mice and chickens vaccinated with these proteins was higher than the nonfused forms of sM2, the GST-C3d-L1-C3d-L2-sM2 groups have conferred the highest 30% and 20% protection ratio in mice and chickens respectively. In addition, the pcDNA-C3d-L1-C3d-L2-sM2 also enhances the antibody responses to sM2 compared to pcDNA-sM2 in chickens, and acquired 13.3% protection ratio.

**Conclusion:**

These results indicated that chicken C3d enhanced the humoral immunity against AIV M2 protein either fused proteins expressed by the prokaryotic system or with the DNA vaccine. Nevertheless, in view of the poor protection ratio for these animals, we speculated that this is not a worthy developing of vaccine in these constructs.

## Background

Complement is a protein system in the plasma of humans and animals [1]. After being activated, a series of important biological reactions generate several complement proteins that nonspecifically defend against invading pathogens [2]. While complement protein C3 is a central component of the innate immune system, it also plays an important role in stimulating the humoral immune response [1,3]. At the point of convergence of three distinct pathways of complement activation, C3 is cleaved into C3a and C3b by the C3 convertase [4]. Further proteolytic cleavage of C3b results in the formation of C3c and C3dg. The C3dg product can be further degraded by a variety of cellular proteases into C3d, a protein which attaches covalently to the surface of pathogens and upregulates B-cell responses [4,5]. Previous studies have demonstrated that C3d could enhance antigen recognition and specific immunoglobulin synthesis by antigen-specific B cells, as the antigen is taken up and processed via cell receptor 2 (CR2) by both antigen-specific and non-specific B cells [6]. Subsequent investigations showed that three copies of murine C3d could dramatically enhance antibody responses to specific antigen, being 100-fold more effective than incomplete Freund's adjuvant [7,8]. Ross reported that C3d could enhance antibody responses directed toward a specific antigen encoded by a DNA vaccine [9]. A DNA vaccine expressing a fusion of hemagglutinin (HA) from influenza virus or measles virus fused to three copies of the murine homologue of C3d (mC3d) achieved an early and efficient immune response in mice. Fusion to C3d has been shown to increase the immunogenicity of the capsular polysaccharide antigen of *Streptococcus pneumoniae *[10]. Using DNA vaccination, various forms of envelope (Env) proteins of the human immunodeficiency virus type 1 (HIV-1) fused at the carboxyl terminus with C3d of murine complement, generated high-titer, long-lasting, neutralizing antibodies in mice [11]. In addition, the human homologue of C3d (hC3d) also enhanced anti-Env antibodies in rabbits when it was fused to sgp120 [12]. Recently, Wang reported that the bovine homologue of C3d (boC3d) coupled to the E2 envelope protein of bovine viral diarrhea virus greatly enhanced immunogenicity in mice [13]. Liu also reported that chicken C3d-P29 linked to the F gene of Newcastle disease virus (NDV) enhanced immunogenicity in chickens [14]. Logan GJ found C3d (3)-fusion markedly increase antibody responses to the AAV-encoded model antigen (hen egg lysozyme) with greater than 50-fold enhancement in responses [15]. Comparison of the human, mouse and bovine C3d sequences showed 84.1% amino acid homology between hC3d and mC3d and 80.5% homology between hC3d and boC3d, they either showed the function of immune adjuvant in mammalian model. Information on the function of avian C3d is scarce. Importantly, there are structural differences in the mammalian and avian immune systems, particularly the role of the bursa as one of the central immune organs in avian species.

Avian influenza (AI), caused by avian influenza virus (AIV), is a highly contagious disease of birds. Current AI vaccines induce antibodies against HA and neuraminidase (NA), two major surface glycoproteins expressed on the virus particles. However, due to rapid antigenic variation of HA and NA, AI vaccine can not protect avian against the new avian influenza virus strains. A vaccine that is less sensitive to the antigenic evolution of the virus would be a major improvement. As a result, vaccines have to be updated continuously to prevent disease emerging due to new viral strains. Hence, a vaccine that could induce broad cross protection against AIV would be desirable.

The Matrix protein 2 (M2) is an integral tetrameric membrane protein of AIV. Natural M2 protein is present in a few copies in the virus particle but in abundance on virus-infected cells. In contrast to hemagglutinin and neuraminidase, M2 is almost nonimmunogenic, and its sequence is highly conserved in all diverse subtypes of AIV. Several investigations have shown that the M2 protein has the potential to induce a broadly protective immunity against AIV. M2-specific antibodies have been shown to restrict virus growth in vitro and in vivo and thus have the potential of providing cross-reactive resistance to influenza type A virus infection [16]. Frace reported that vaccination with the protein M2 was found to raise M2-specific serum antibodies and enhance viral clearance in mice challenged with homologous and heterologous influenza A viruses [17]. Neirynck reported that the M2 domain was genetically fused to the hepatitis B virus core (HBc) protein to create fusion gene coding for M2HBc, and intraperitoneal or intranasal administration of purified M2HBc particles to mice provided 90-100% protection against a lethal virus challenge [18]. Zhao designed a tetra-branched multiple antigenic peptide (MAP)-based vaccine, designated M2e-MAP, which contains the sequence overlapping the highly conserved extracellular domain of matrix protein 2 (M2e) of a HPAI H5N1 virus, animals test results showed that M2e-MAP vaccine induced strong M2e-specific IgG antibody responses following 3-dose immunization of mice with M2e-MAP in the presence of Freunds' or aluminium (alum) adjuvant [19]. Rao SS have shown that vaccination with M2 in recombinant DNA and/or adenovirus vectors or with adjuvants confers protection against lethal challenge in the absence of HA, and also find that the protective efficacy of NP and M2 diminishes as the virulence and dose of the challenge virus are increased [20]. However, M2 is only a minor protein component of AIV, and tends to induce a poor immune response [21]. To overcome the shortcomings of M2-based vaccines, in this study we investigated the potential of M2-avian C3d fusion proteins to provide effective immunity.

## Results

### Cloning and sequence analysis of the chicken C3d gene fragment

**Figure 1:**
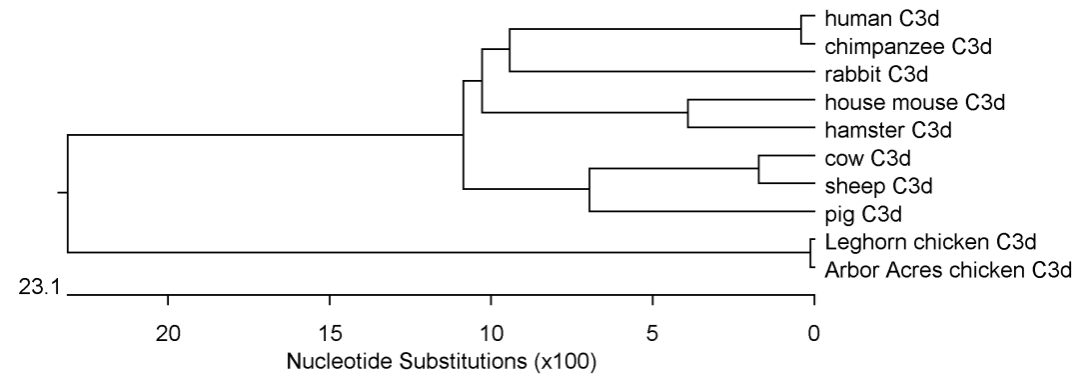
**Phylogenetic tree of the C3d amino acid sequences from chicken and other species**. The phylogenetic tree was generated by neighbour-joining analysis with Clustal W, using DNAStar.

Chicken C3d has not previously been cloned so it was necessary to clone and sequence the complement C3d fragment. A PCR product of approximately 1 kb was amplified from RNA extracted from chicken liver tissue. The fragment was cloned into the pMD18-T (Takara) plasmid and sequenced. Sequence analysis revealed that the amplified fragment was 993 bp in length and that the chicken C3d gene was 897 bp in length. This nucleotide sequence for chicken C3d was submitted to the GenBank database (DQ291160). The chicken C3d sequence showed the following % identities to the published sequences for C3d from: Arbor Acres chicken (EF632299) 99.8%; human (NM_000064) 66.4%; mouse (BC043338) 66.2%; hamster (AB024425) 67.9%; cow (AY630404) 67.2%; rabbit (M32434) 67.0%; pig (NM_214009) 66.8%; chimpanzee (XM_512318) 66.4% and sheep (AF038130) 66.9%, as determined by Clustal W multiple alignment of nucleotide sequences. The predicted protein sequence of chicken C3d showed the following % similarity to the published protein sequences for C3d from: Arbor Acres chicken, 100%; human, 61.5%; mouse, 61.9%; hamster, 61.2%; cow, 62.2%; rabbit, 56.5%; pig, 61.9%; chimpanzee, 61.5% and sheep, 61.5%, as determined by DNAStar ClustalW analysis. A phylogenetic tree of the C3d amino acid sequences is shown in Fig. 1.

### Construction and identification of the recombinant expression plasmids

Recombinant expression plasmids pGEX-C3d-sM2, pGEX-C3d-L1-C3d-sM2, pGEX-C3d-L2-sM2, pGEX-C3d-L1-C3d-L2-sM2 and pcDNA-sM2, pcDNA-C3d-L1-C3d-L2-sM2 were constructed with gene expression cassettes, pGEX-5x-1 and pcDNA4.0-*LacZ*. *Eco*RI and *Xho*I restriction endonuclease digestion resulted in a linearized pGEX-5x-1plasmid (of about 4900 bp), expression cassettes DNA of two copies of cC3d and sM2 (of about 2100 bp) and gene cassettes DNA of one copy of cC3d and sM2 (of about 1200 bp).

### Expression and purification of fusion proteins

**Figure 2:**
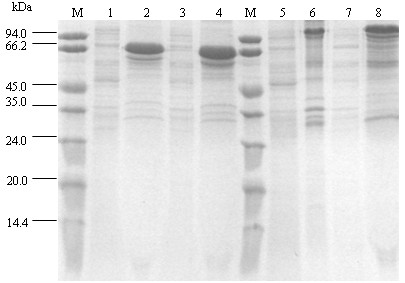
**SDS-PAGE analysis of proteins expressed by the expression vectors containing one or two copies of C3d fused to sM2 in E. coli, before and after induction**. M: protein marker; lane 1: pGEX-C3d-L2-sM2 before induction; lane 2: pGEX-C3d-L2-sM2 after induction; lane 3: pGEX-C3d-sM2 before induction; lane 4: pGEX-C3d-sM2 after induction; lane 5: pGEX-C3d-L1-C3d-sM2 before induction; lane 6: pGEX-C3d-L1-C3d-sM2 after induction; lane 7: pGEX-C3d-L1-C3d-L2-sM2 before induction; lane 8: pGEX-C3d-L1-C3d-L2-sM2 after induction.

*E. coli *BL21 (DE3) cells were transformed with the four prokaryotic expression plasmids detailed above. Cells were then induced by IPTG at 28°C. The expression products were purified using the SKL method. Examination by 12% SDS-PAGE showed that the molecular weight of the single fused copy of cC3d was about 70 kDa, while the molecular weight of the two copies fused to cC3d was about 102 kDa, both of these sizes corresponding to the sizes expected (Fig. 2).

### SDS-PAGE and Western immunoblot detection of fusion proteins

**Figure 3:**
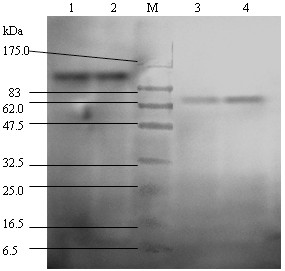
**Western-blot analysis of the recombinant proteins GST-C3d-L1-C3d-L2-sM2, GST-C3d-L1-C3d-sM2, GST-C3d-L1-sM2 and GST-C3d-sM2**. M: protein marker; lane 1: GST-C3d-L1-C3d-sM; lane 2: GST-C3d-L1-C3d-L2-sM2; lane 3: GST-C3d-sM2; lane 4: GST-C3d-L1-sM2.

Western blot analysis was performed with purified GST-C3d-L1-C3d-L2-sM2, GST-C3d-L1-C3d-sM2, GST-C3d-L1-sM2 and GST-C3d-sM2 proteins and the anti-sM2 monoclonal antibody, which had been prepared in our laboratory. The band of interest was evident on Western blot analysis, indicating that the recombinant protein had maintained the antigenicity of the sM2 component (Fig. 3).

### Expression of recombinant eukaryotic plasmids in BHK-21 cells

**Figure 4:**
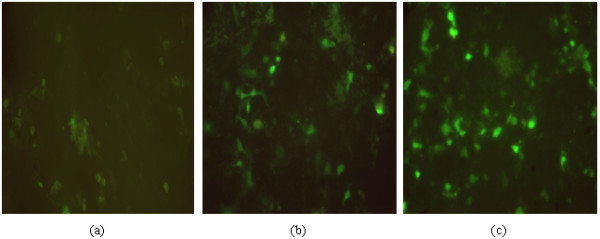
**Expression in BHK-21 cells of pcDNA-sM2 and pcDNA-C3d-L1-C3d-L2-sM2 as detected by IFA**. (a) Transfection of pcDNA (200×); (b) Transfection of pcDNA-sM2 (200×); (c) Transfection of pcDNA-C3d-L1-C3d-L2-sM2 (200×).

The constructed eukaryotic expression vectors pcDNA-sM2, pcDNA-C3d-L1-C3d-L2-sM2 and pcDNA4.0 were transfected into BHK-21 cells. The indirect immunofluorescence test demonstrated that the sM2 protein was expressed in BHK-21 cells after the cells had been transfected with the pcDNA-sM2 and pcDNA-C3d-L1-C3d-L2-sM2 eukaryotic expression plasmids (Fig. 4).

### Chicken C3d increases the antibody titers of sM2 in BALB/c mice and protection against challenge

**Figure 5:**
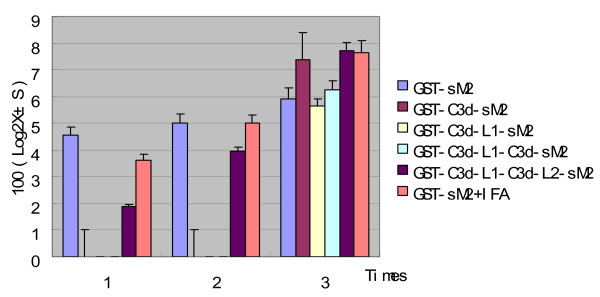
**Titers of antibody against sM2 in BALB/c mouse serum detected by ELISA**. Sixty BALB/c mice aged 6 weeks were divided into 6 (n = 10) groups. The mice in the six test groups were immunized thrice with GST-sM2, GST-C3d-sM2, GST-C3d-L1-sM2, GST-C3d-L1-C3d-sM2, GST-C3d-L1-C3d-L2-sM2 and GST-sM2+IFA, respectively. Blood samples were collected before each vaccination and two weeks after the final booster, and antibody against sM2 were detected by ELISA. Ab-positive cut-off values were set as mean + 2 SD of non-immunized sera. An ELISA Ab titer was expressed as the highest serum dilution giving a positive reaction. The titer values were showed in 100 (Log2X ± S).

BALB/c mice were immunized three times with purified GST-C3d-L1-C3d-L2-sM2, GST-C3d-L1-C3d-sM2, GST-C3d-L1-sM2, GST-C3d-sM2, GST-sM2 proteins and GST-sM2 mixed in IFA. The results showed that no anti-sM2 antibody was produced after the first and second immunizations with GST-C3d-L1-C3d-sM2, GST-C3d-L1-sM2 and GST-C3d-sM2 (Fig. 5). After the third immunization, high level sM2 antibody titers appeared in six immunized groups. The difference between the GST-C3d-sM2, GST-C3d-L1-C3d-L2-sM2, GST-sM2 mixed in IFA immunized groups and the control group (which was GST-sM2) was very significant (P < 0.01), the anti-sM2 antibody in mice primed with GST-C3d-L1-C3d-L2-sM2 was greater than that in GST-sM2 mixed in IFA group, but the difference between the two groups was not significant (P > 0.05). The GST-sM2+IFA and GST-C3d-L1-C3d-L2-sM2 groups have conferred the highest 30% protection ratio in mice (Table 1), the GST-C3d-sM2 group provide 20% protection ratio secondly. Most of mice that attacked with H9N2 virus have showed some clinical signs after post-challenge, such as depressed, shiver and so on.

**Table 1:** Results of protection from immunized mice after challenge.

Groups	Vaccine	Number of positive/total	Protection ratio (%)
1	GST-sM2	10/10	0
2	GST-C3d-sM2	8/10	20
3	GST-C3d-L1-sM2	9/10	10
4	GST-C3d-L1-C3d-sM2	9/10	10
5	GST-C3d-L1-C3d-L2-sM2	7/10	30
6	GST-sM2+IFA	7/10	30

Mice were immunized thrice with GST-sM2, GST-C3d-sM2, GST-C3d-L1-sM2, GST-C3d-L1-C3d-sM2, GST-C3d-L1-C3d-L2-sM2 and GST-sM2+IFA, respectively. Then all the mice were challenged with 10^6.5 ^EID_50 _of the A/chicken/Bei Jing/WD9/98 (H9N2) virus. On the indicated days, mice were euthanatized, lung samples were collected and subsequently homogenized and inoculated in SPF chicken embryos for the presence of infectious virus. Virus isolation was operated as described in the Materials and Methods. The mouse was considered to be protected whichever HA test was negative in two virus isolations. The protections from immunized mice on day 5 p.i. are shown.

### Chicken C3d enhances the antibody titers of sM2 in SPF chickens and protection against challenge

**Figure 6:**
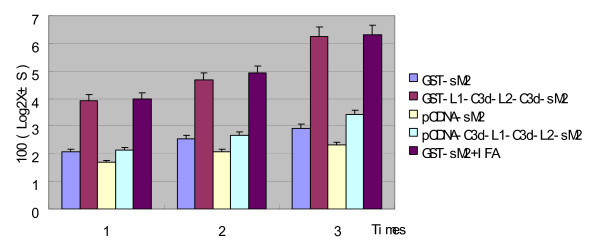
**Titers of antibody against sM2 in SPF chicken serum detected by ELISA**. Seventy-five SPF chickens aged 3 weeks were divided into 5 (n = 15) groups. The chickens in the five test groups were immunized thrice with GST-sM2, GST-C3d-L1-C3d-L2-sM2, pcDNA-sM2, pcDNA-C3d-L1-C3d-L2-sM2 and GST-sM2+IFA, respectively. Blood samples were collected before each vaccination and two weeks after the final booster, and antibody against sM2 were detected by ELISA. Ab-positive cut-off values were set as mean + 2 SD of non-immunized sera. An ELISA Ab titer was expressed as the highest serum dilution giving a positive reaction. The titer values were showed in 100 (Log2X ± S).

SPF chickens were immunized three times with GST-C3d-L1-C3d-L2-sM2, GST-sM2, GST-sM2 mixed in IFA and DNA vaccines of pcDNA-sM2 and pcDNA-C3d-L1-C3d-L2-sM2. In the indirect ELISA, for the fusion protein groups, the difference between GST-C3d-L1-C3d-L2-sM2, GST-sM2 mixed in IFA and GST-sM2 (control group) was very significant (P < 0.01) after the second immunization. Indeed, the antibody titer of the GST-C3d-L1-C3d-L2-sM2 immunized group was higher than that of all the other groups at this point. The difference between GST-C3d-L1-C3d-L2-sM2 and GST-sM2 mixed in IFA group was not significant (P > 0.05) after the second or third immunization. After the third immunization, the difference between the experimental pcDNA-C3d-L1-C3d-L2-sM2 group and the control pcDNA-sM2 group became significant (P < 0.05) (Fig. 6). The GST-sM2+IFA group has the highest antibody titer and a 26.7% protection ratio (Table 2), the GST-C3d-L1-C3d-L2-sM2 and pcDNA-C3d-L1-C3d-L2-sM2 groups provide 20%, 13.3% protection ratio respectively. No chicken that received H9N2 virus have showed obvious clinical signs on 5 days post challenge.

**Table 2:** Results of protection from immunized chickens after challenge.

Groups	Vaccine	Number of positive/total	Protection ratio (%)
1	GST-sM2	0/15	0
2	GST-C3d-L1-C3d-L2-sM2	3/15	20
3	pcDNA-sM2	1/15	6.7
4	pcDNA-C3d-L1-C3d-L2-sM2	2/15	13.3
5	GST-sM2+IFA	4/15	26.7

Chickens were immunized thrice with GST-sM2, GST-C3d-L1-C3d-L2-sM2, pcDNA-sM2, pcDNA-C3d-L1-C3d-L2-sM2 and GST-sM2+IFA, respectively. Then all the Chickens were challenged with 5× 10^6.5 ^EID_50 _of the A/chicken/Bei Jing/WD9/98 (H9N2) virus. On the indicated days, chickens were euthanatized, lung samples were collected and subsequently homogenized and inoculated in SPF chicken embryos for the presence of infectious virus. Virus isolation was operated as described in the Materials and Methods. The chicken was considered to be protected whichever HA test was negative in two virus isolations. The protections from immunized mice on day 5 p.i. are shown.

### Lymphocyte transformation assay (MTT method) of SPF chickens

After the third immunization, peripheral blood lymphocytes were isolated from the SPF chickens and used in the lymphocyte transformation assay (see Table 3). Statistical analysis of the OD_570 _values showed that with respect to LPS-stimulated lymphocytes, the difference in OD values between the group immunized with GST-C3d1-L1-C3d2-L2-sM2 fusion protein and control group immunized with GST-sM2 was very significant (P < 0.01). The difference between the group immunized with pcDNA-C3d-L1-C3d-L2-sM2 plasmid and the control group immunized with pcDNA-sM2 was also very significant (P < 0.01). The lymphocytes in each group stimulated with Con A showed no significant differences.

**Table 3:** Results of the lymphocyte transformation test.

Groups	**Mean OD value**^a^	
	
	LPS	Con A
pcDNA-sM2	0.163 ± 0.011^A^	0.226 ± 0.015^A^
GST-sM2	0.283 ± 0.009^AC^*	0.272 ± 0.013^A^
pcDNA-C3d-L1-C3d-L2-sM2	0.533 ± 0.012^B^**	0.314 ± 0.011^A^
GST-C3d-L1-C3d-L2-sM2	0.962 ± 0.023^D^**	0.321 ± 0.011^A^
GST-sM2+IFA	0.981 ± 0.023^E^**	0.318 ± 0.011^A^

A, B, C, D and E indicate a significant difference between each group. A value represent means ± SD of each group (n = 15). *Significantly different from the control groups (p < 0.05) by ELISA. **Significantly different from the control groups (p < 0.01) by ELISA.

## Discussion

Many researchers have confirmed that in the immune system of mammals, complement component C3d as a molecular adjuvant bridging innate and acquired immunity [7,22,23]. Apart from enhancing the activation of B cells, promoting the affinity maturity of antibodies and maintaining immunologic memory, C3d also has the molecular adjuvant function of enhancing the antigen presentation of B cells as well as decreasing the activation threshold of these cells [24-26]. As a molecular adjuvant, C3d can be linked to different antigens, effectively enhancing the titer and life-span of specific antibodies against these antigens [7,27-29]. However, there have been few reports about the structure and function of chicken C3d published to date. In 2005, we cloned the full-length chicken *C3d *gene and submitted its sequence to the GenBank database (Accession number: DQ291160). Amino acid homology between chicken C3d and mammal C3d was found to be low.

In this study, we fused one to two copies of C3d to the 5' end of *sM2 *gene of AIV to construct expression plasmids, from which fusion proteins were expressed and purified. We immunized BALB/c mice and SPF chickens with the purified proteins. The experimental results in mice and chickens showed that C3d could enhance the antibody titer to sM2 protein in both host species. In studies using human and mouse C3d, most researchers considered that at least three copies of C3d fused to antigen were needed to enhance immunity [30-32]. Suradhat also reported that two copies of mouse C3d fused to bovine rotavirus (BRV) VP7 or bovine herpesvirus type I (BHV-I) glycoprotein D did not enhance antibody titers to either antigen [33]. Bower reported that there was no relationship between the copy numbers of C3d binding to antigen and the antibody titer [34]. In our present study, no obvious changes in antibody levels were detected in each C3d injected group after the first and second immunizations, so repeated immunizations were performed to improve the immune response. However, after the third immunization, both one copy and two copies of C3d significantly enhanced the antibody titer against sM2. This indicates that repeated immunization with chicken C3d can enhance the humoral immune response to an antigen molecule. The antibody titers of sM2 produced by immunizing the fusion proteins of two copies of C3d and sM2 bound by two simple amino acid flexible peptides, are significantly higher than for other treatment groups. This result was further verified by immunization of SPF chickens, and agreed with the findings of a previous report [35]. It is not clear why in mice immunized with fusion proteins bearing one copy of C3d or with fusion proteins that consisted of two copies of C3d only linked with one flexible peptide, anti-sM2 antibody could not be detected after two immunizations. It is possible that, because the molecular weight of C3d is greater than that of sM2, the C3d stereochemistry blocked sM2 binding. In contrast, with the protein GST-C3d-L1-C3d-L2-sM2, which has two flexible peptides, repeated immunizations are not required, a finding that has been reported previously [34-36].

Many previous researches have shown the molecular adjuvant effect of C3d in the immunization of DNA vaccines [33,36-39]. However, our immunological test in SPF chickens found that C3d can enhance the immunogenicity of the linked sM2 protein, whether it exists as a prokaryotic expressed fusion protein or a eukaryotic recombinant plasmid. The reason for this was not clear. We also found that DNA vaccine bearing two copies of C3d can enhance the immunogenicity of the antigen sM2, but its ability to increase the antibody titer is lower than that of the fusion proteins.

The transformation ability of lymphocytes may reflect the immune status of the body. Mitsuyoshi reported that C3d might enhance the immunogenicity of antigens without T lymphocytes [40]. In the present study, we used LPS and Con A as mitogens to perform peripheral blood lymphocyte transformation tests. The results showed that when stimulated with LPS, the differences between the GST-C3d-L1-C3d-L2-sM2 immunized group and the matching control group and between the pcDNA-C3d-L1-C3d-L2-sM2 immunized group and the matching control group were significant. However, when stimulated with Con A, there was no significant difference between the immunized groups. Since LPS and Con A are major mitogens of B and T lymphocytes respectively, these findings indicate that C3d can enhance the proliferation of chicken B lymphocytes but has no obvious effect on the proliferation of T lymphocytes. In contrast, Mitchel reported that C3d could activate T cells and express major histocompatibility complex class I and II proteins in an antigen dependent manner [29]. Also, Bower reported that C3d could induce higher expression of T cells stimulating secretions of interferon-γ and interleukin-4 [41]. Based on this research, our future studies will focus on the effect of chicken complement component C3d on cell-mediated immunity.

In this review, though the results presented here suggest that C3d as an adjuvant might be effective to enhance antibody response, a good AIV vaccine is the ability to protect vaccined animals. In order to improve the vaccine efficacy, one or two copies of chicken *C3d *and flexible peptides were applied in the constructs of vaccines, but the M2 vaccines still needs at least two booster immunizations. Furthermore, Vaccinations as described here, only afforded 30% protection in mice and 20% protection in chickens. Therefore, this is not a promising vaccine in these constructs. We hope that it is helpful to develop a good universal AIV vaccine based on the M2 protein.

## Conclusion

In this study, we investigated the potential of M2-avian C3d fusion proteins to provide effective immunity. These results indicated that chicken C3d enhanced the humoral immunity against AIV M2 protein either fused proteins expressed by the prokaryotic system or with the DNA vaccine. Nevertheless, in view of the poor protection ratio for these animals, we speculated that this is not a worthy developing of vaccine in these constructs.

## Methods

### Cloning and sequencing of the chicken C3d gene fragment

Using the publicly available sequences of human, mouse, hamster, cow, rabbit, pig, chimpanzee and sheep complement component C3d and the fowl complement component C3, two primers (upstream: 5'-CCT GGT GGA GAA AGC C-3'; downstream: 5'-TGC GGT AGG TGA TGG C-3') were designed. Samples of liver tissue were obtained from a specific-pathogen-free (SPF) White Leghorn chicken inoculated with a 0.5 ml dose of infectious coryza oil vaccine. RNA was extracted using Trizol reagent (Invitrogen, USA) as per the manufacturer's instructions. A reverse transcriptase (RT) reaction was carried out using the above reverse primer and Maloney murine leukemia virus reverse transcriptase (Invitrogen, USA) to generate cDNA containing the chicken complement C3d sequence. The C3d gene was PCR amplified using the above cDNA as a template. Standard PCR conditions consisted of an initial denaturation step at 94°C (5 min), followed by 30 cycles of denaturing at 94°C (30 sec), annealing at 56°C (45 sec) and extending at 72°C (1 min), followed by a 10 min final extension step at 72°C. The plasmid sequence was verified by automated nucleotide sequence analysis using standard protocols. The RT-PCR product was inserted into pMD18-T (Takara) by TA-cloning to facilitate subsequent manipulation, and the recombined plasmid was designated pMD-cC3d.

### Construction and identification of the recombinant expression plasmids

Primers were designed for PCR amplification of the full-length gene sequence of (918 bp) chicken C3d (cC3d) from the sequence of the recombinant plasmid pMD-cC3d. The forward primer (5'-**GAATTC **ATGCACCTCATTGTGACCCCCTCGGGCAGT-3') was specific for the 5' coding region of the chicken *C3d *gene and began with an upstream *Eco*RI restriction site (in bold) and start code. The reverse primer (5'-**CCCGGG**GCGGTAGGTGATGGCGTTG-3') coded for the 3' region and provided an *Xma*I restriction site (in bold). The *sM2 *gene of AIV (A/chicken/Guangdong/2000, H9N2) was amplified from plasmid pMD-sM2 by PCR [42]. The amplified *sM2 *gene fragment (256 bp in length) encoded the complete M2 open reading frame of AIV but not the transmembrane domain. Upstream *Hind*III and downstream *Xho*I sites were added to the sM2 fragment. Two linker sequences (L1 and L2) were constructed following the design of Dempsey et al. [7] to provide spacing between protein motifs. Four single stranded oligonucleotides encoding the amino acid sequence GS [G_4_S]_2_GS with upstream *Xma*I and downstream *Hind*III or *Nhe*I sites were prepared by forming adhesive ends after annealing. One or two copies of the *cC3d *and *sM2 *genes were joined with different linkers to form four gene expression cassettes: *EcoR*I-C3d-*Hind*III-sM2-*Xho*I, *Eco*RI-C3d-*Xma*I-L2-*Hind*III-sM2-*Xho*I, *Eco*RI-C3d-*Xma*I-L1-*Nhe*I-C3d-*Hind*III-sM2-*Xho*I and *EcoR*I-C3d-*Xma*I-L1-*Nhe*I-C3d-*Xma*I-L2-*Hind*III-sM2-*Xho*I. To construct the plasmids needed, these gene expression cassettes were ligated into *EcoR*I and *Xho*I sites of a plasmid vector pGEX-5x-1 (Amersham-Pharmacia). The gene cassette encoding *Eco*RI-C3d-*Xma*I-L1-*Nhe*I-C3d-*Xma*I-L2-*Hind*III-sM2-*Xho*I was cloned into the identical site of the eukaryotic plasmid pcDNA4.0-Lacz (Invitrogen) containing the cytomegalovirus immediate-early promoter, an upstream intron and the simian virus 40 late poly (A) signal sequences. As a control, the *sM2 *gene alone was inserted into plasmids pGEX-5x-1 and pcDNA4.0-Lacz respectively to construct recombinant expression plasmids [43,44].

### Expression and purification of fusion proteins

The recombinant prokaryotic expression plasmids were transformed into competent *Escherichia coli *strain BL21 (DE3). Single colonies of four positive recombinant bacteria were inoculated into Luria broth (LB) supplemented with 100 μg/ml ampicillin. These cultures were incubated at 37°C until mid-log phase (OD_600 _0.6-0.8). The broth was then divided into two, with one half being induced with 1 mM isopropyl β-D-thiogalactoside (IPTG) while the other half served as an uninduced control. The broths were incubated for an additional 4 h at 28°C. The expression product was purified from the supernatants of bacterial lysates by affinity chromatography using glutathione sepharose 4B (Amersham-Pharmacia). Protein concentrations were determined with a Bio-Rad protein assay dye reagent concentrate, and the purified proteins were stored at -80°C.

### SDS-PAGE and Western immunoblot detection of fusion proteins

Expressed proteins were separated by electrophoresis in 12% sodium dodecyl sulfate (SDS)-polyacrylamide gels. To visualize total proteins, gels were stained with Bio-Safe™ Coomassie G250 stain (New England Biolabs). Protein All Blue™ standard (New England Biolabs) was used as a molecular mass standard.

For Western immunoblot analysis, proteins were transferred to nitrocellulose membranes (Millipore). The membranes were blocked for a minimum of 1 h with PBS containing 0.5% bovine serum albumin (BSA) (Sigma) and 0.1% Tween 20 (Sigma) prior to incubation with a 1:500 dilution of a monoclonal antibody (3F8) specific for sM2 (primary antibody, which is prepared in previous research by ourselves). After extensive washing, bound antibodies were detected by enhanced chemiluminescence (Amersham) with a 1:5000 dilution of horseradish peroxidase-conjugated anti-mouse IgG (Sigma) (secondary antibody). Blots were developed using alkaline phosphatase (AP) Conjugate Substrate Kit (New England Biolabs). Color development was stopped by washing the membrane in deionized water.

### Expression of recombinant eukaryotic plasmids in BHK-21 cells

The eukaryotic plasmids containing *Eco*RI-C3d-*XmaI*-L1-*Nhe*I-C3d-*Xma*I-L2-*Hin*dIII-sM2*-Xho*I and the sM2 gene alone were isolated from *E. coli *DH5á cells and purified using a Plasmid Mini Purification Kit (QIAGEN GmbH). The endotoxin-free plasmids were verified by restriction endonuclease digestion with *Eco*RI and *Xho*I and analysis by gel electrophoresis. The purity and concentration of DNA preparations was determined based on the optical densities (ODs) at 260 and 280 nm. In a six-well plate (Costar), baby hamster kidney cell lines (BHK-21) on slides were transfected with 4.0 μg of DNA and 10 μl of Lipofectamine™ 2000 (Life Technologies, Grand Island, N. Y.) according to the manufacturer's guidelines. The transfected cells were incubated for 36 h at 37°C in a CO_2 _incubator, and then harvested for immunological examination.

### Indirect immunofluorescence detection

The slides that carried the infected cells were carefully removed from the wells, washed with 0.01 M PBS, then fixed (acetone: absolute alcohol, 3:2) for 30 min at 4°C. The fixed BHK-21 cells were incubated with a 1:100 dilution of monoclonal antibody (3F8) against sM2 for 45 min at 37°C in a wet box. After extensive washing, bound antibodies were detected using a FITC-conjugated goat anti-mouse IgG (Sigma) and observed by fluorescence microscopy.

### Animals immunization and challenge

Six-week-old BALB/c mice (Jingfeng Medical Laboratory Institute, Beijing, China) were inoculated. Mice were randomly divided into six groups, with 10 mice in each group, and housed with free access to food and water. Mice were administered with purified proteins GST-sM2, GST-C3d-sM2, GST-C3d-L1-sM2, GST-C3d-L1-C3d-sM2 and GST-C3d-L1-C3d-L2-sM2 in 100 μl of PBS subcutaneously in the rear flank. Mice were also injected with GST-sM2 in incomplete Freund's adjuvant (IFA; Sigma) as control group. Booster immunizations with the same proteins were performed with an interval of two weeks. For the three inoculations, the injection dose was 100 μg proteins respectively. Non-lethal tail bleeds were collected before each vaccination and two weeks after the final booster. For virus challenge, phenobarbital sodium-anesthetized mice were intranasally infected with A/chicken/Bei Jing/WD9/98 (H9N2) virus (10^6.5 ^EID_50 _[the 50% embryo infectious dose]) in 100 μl of PBS per mouse 2 weeks after the final immunization. Lung samples were collected from individual mice at day 5 after a challenge infection. The whole-lung extracts prepared as homogenates using frosted glass slides were centrifuged at 3,000 rpm for 5 min to collect supernatants. The lung supernatants were frozen and kept at -70°C until used for virus isolation assay. The supernatants (0.2 ml/egg) were inoculated into the allantoic cavity of five 10-days old specific pathogen free (SPF) chicken embryos. Early embryonic death within the first 24 hours of inoculation was considered as non-specific death and these embryos were discarded. After incubation at 37°C for 5 days the allantoic fluid was harvested and tested by haemagglutination (HA) assay. In the cases there was no virus was detected in the first virus isolation, the allantoic fluid was passaged once in embryonated hen eggs. The mouse was considered not to be protected whichever HA test was positive in two virus isolations. The mouse was considered to be protected when the second passage HA test still was negative.

75 aged 3 weeks SPF chickens (Beijing Center for Laboratory Animals, Beijing) were divided into five equal groups. Three groups of chickens were injected intramuscularly with 200 μg of purified proteins GST-sM2, GST-sM2 mixed with incomplete Freund's adjuvant (IFA; Sigma) and GST-C3d-L1-C3d-L2-sM2 respectively. The other two groups were vaccinated with the mixtures of the recombinant plasmids DNA (pcDNA-sM2 and pcDNA-C3d-L1-C3d-L2-sM2) and Lipofectamine™ 2000 (Invitrogen) by the intramuscular route. A dose of 0.2 ml contained 100 μg DNA and 50 μl Lipofectamine™ 2000 in Opti-MEM^® ^I Reduced Serum Medium (Invitrogen). Two booster immunizations of the same purified proteins and plasmid DNA were given at an interval of two weeks, with the same dose being 100 μg respectively. Blood was collected from the wing vein before every vaccination and two weeks after the final boost. Virus challenge and virus isolation trials were carried out refer to the above mouse test procedure, 5×10^6.5 ^EID_50 _of H9N2 virus was used as the challenge viral dosage. All of the animal experiments performed in this study were approved by the Beijing Laboratory Animal Management Office (China).

### Indirect ELISA for detection of anti-sM2 antibodies

According to previously described methods [15,43], purified sM2 protein was obtained by cleaving GST-sM2 bound to Glutathione Sepharose 4B (Amersham-Pharmacia) with factor Xa. Polystyrene microplates (Costar, Corning Incorporated) were coated with 100 μl of purified sM2 protein at a concentration of 0.125 μg/ml, and the samples were incubated with 2-fold serially diluted sera from the vaccinated animals. Anti-mouse (or anti-chicken) IgG conjugated to horseradish peroxidase (Sigma, 1:8000 dilutions) was used as the secondary antibody. End-point titers were calculated as the reciprocal of the last serum dilution that gave a value 2-fold higher than the negative serum. Antibody titers below the cutoff of the assay were assigned an arbitrary titer one-half the cutoff in order to allow calculation of the geometric mean of the titers.

### Lymphocyte proliferation assay

Ten SPF chickens from each of the immunized groups described above were bled aseptically from the heart two weeks after the final boost. Peripheral blood lymphocytes were isolated by Lympholyte ^®^-Mammal (Cedarlane^®^, Laboratories Limited). The cells were resuspended in RPMI 1640 medium supplemented with 10% fetal calf serum, 100 μg/ml penicillin and 100 μg/ml streptomycin (all from Invitrogen). Freshly isolated lymphocytes were seeded in quadruplicate at 4×10^6 ^cells/ml in 96-well round-bottomed microtiter plates (Costar). Cells were either left un-stimulated or stimulated with lipopolysaccharide (LPS; 5 μg/ml; Sigma) or Concanavalin A (Con A; 5 μg/ml; Sigma) as the mitogen. After incubation for 60 h at 37°C, 3- [4, 5-dimethylthiozol-2-yl]-2, 5-diphenylthtrazolium bromide (MTT) was added to the cultures and a further 3 h incubation performed. The reaction was stopped with 10% SDS-0.01 mol/l HCL and the OD_570 nm _was measured using a Bio-Rad 550 plate reader.

### Statistical analysis

The anti-sM2 antibody titers were expressed as the 100 Log_2_X where X was the reciprocal of the final dilution of serum. Proliferation of lymphocytes were expressed as the OD_570 _value which was expressed as the mean ± standard errors of the mean (SEM) of at least three independent experiments. The *t*-test was used to analyze the statistical significance of the differences. Probability (*P*) values of < 0.05 (*) and < 0.01 (**) were considered significant.

## List of abbreviations used

sM2: M2 protein with the transmembrane region deleted; GST: Glutathione S-transferase tagged proteins in pGEX expression vector; ELISA: enzyme-linked immunosorbent assay; SPF: specific pathogen free; CR2: cell receptor 2; HIV-1: human immunodeficiency virus type 1; IPTG: isopropyl β-D-thiogalactoside; BSA: bovine serum albumin.

## Competing interests

The authors declare that they have no competing interests.

## Authors' contributions

ZHZ carried out most of the experiments and wrote the manuscript. YQL and FYC carried out study design, also wrote and revised the manuscript. SFX and LZ helped in experiments, participated in antibody detection and statistical analyses. BYJ and XLC conceived the study, and participated in its design and coordination, also helped to look over the manuscript. All authors read and approved the final manuscript.
